# Supplementary material for: Expression of Neurotrophins and Its Receptors During Fetal Development in the Human Cochlea
Source: Int J Mol Sci. 2024 Dec 3;25(23):13007. doi: 10.3390/ijms252313007 (PMC11641258; doi:10.3390/ijms252313007)
Supplement: Supplementary file 1 [file ijms-25-13007-s001.zip › ijms-3305626-supplementary.pdf]

Supplementary Material

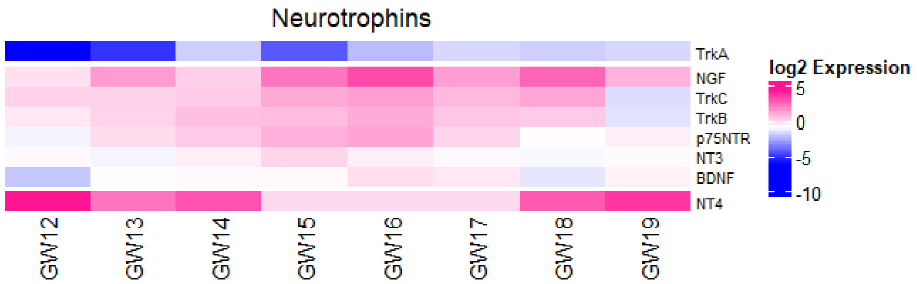

Supplementary Figure S1. Differential expressed (DE) genes identified from the RNAseq data of neurotrophin receptors (TrkA, TrkB, TrkC, $p75^{NTR}$) and their ligands (BDNF, NT-3, NT-4, NGF) genes between GW12 to GW19 relative to GW11. The RNAseq profile is represented as DE expression of differential expressed genes (y-axis) and gestational week (x-axis). GW11 is taken as the experimental calibrator for RNAseq data. Heatmap was generated with Clustering distance row of “Maximum” “Maximum” and Clustering method rows with “Median”.
